# Endemic and Other Dimorphic Mycoses in The Americas

**DOI:** 10.3390/jof7020151

**Published:** 2021-02-20

**Authors:** Shawn R. Lockhart, Mitsuru Toda, Kaitlin Benedict, Diego H. Caceres, Anastasia P. Litvintseva

**Affiliations:** 1 Mycotic Diseases Branch, Centers for Disease Control and Prevention, Atlanta, GA 30329, USA; nrk7@cdc.gov (M.T.); jsy8@cdc.gov (K.B.); xju7@cdc.gov (D.H.C.); frq8@cdc.gov (A.P.L.); 2 Department of Medical Microbiology, Radboud University Medical Center and Center of Expertise in Mycology Radboudumc/Canisius Wilhelmina Hospital, 6525 Nijmegen, The Netherlands

**Keywords:** *Histoplasma*, *Blastomyces*, *Coccidioides*, *Sporothrix*, *Paracoccidioides*, endemic, America

## Abstract

Endemic fungi are thermally dimorphic fungi that have a limited geographic range and can cause both primary disease and opportunistic infections. The Americas are home to more genera of endemic fungi than anywhere else on earth. These include *Coccidioides*, *Histoplasma*, *Blastomyces*, *Paracoccidioides,* and *Sporothrix*. Endemic fungi are found across the Americas and the Caribbean, from *Blastomyces gilchristi,* which extends into the northeast corners of North America, to *Histoplasma capsulatum,* which occurs all the way down in the southern regions of South America and into the Caribbean Islands. Symptoms of endemic fungal infection, when present, mimic those of many other diseases and are often diagnosed only after initial treatment for a bacterial or viral disease has failed. Endemic fungi place a significant medical burden on the populations they affect, especially in immunocompromised individuals and in resource-limited settings. This review summarizes the ecology, geographical range, epidemiology, and disease forms of the endemic fungi found in the Americas. An emphasis is placed on new and proposed taxonomic changes, including the assignment of new species names in *Histoplasma*, *Blastomyces,* and *Paracoccidioides*.

## 1. Introduction

The Americas contain the largest number of dimorphic endemic fungal species, including *Coccidioides* species, *Histoplasma capsulatum*, *Blastomyces* species, *Paracoccidioides* species, *and Sporothrix* species. The endemic fungi are so named because they have regional endemicity rather than being cosmopolitan like most fungi. They also share the trait of being thermally dimorphic, growing as a mold in the environment at temperatures below 37 °C and as a yeast [or spherule in the case of *Coccidioides*] in the host. Unlike most other fungal pathogens, which primarily infect people with weakened immune systems, the endemic fungi can act as primary pathogens and cause infections in people with intact immune systems. Conidia for all the endemic fungi are present intermittently in their respective endemic areas, but environmental disruption is often a key factor in their dispersal and likelihood of causing infection. Because endemic fungi are rarely cultured from environmental specimens, much remains unknown about their environmental niches and geographic ranges.

Signs and symptoms of endemic fungal diseases can be nonspecific and similar to those of other respiratory diseases, particularly other types of pneumonia [1]. Therefore, laboratory testing is needed to detect these fungal diseases. However, low healthcare provider awareness, lack of clear diagnostic guidance, unavailability of diagnostic testing in some areas, and the fact that some fungal diagnostic test results can be challenging to interpret all suggest that these diseases are likely widely underdiagnosed. The lack of comprehensive surveillance for most of these diseases also means that they are underreported to public health authorities, leaving a gap in our understanding of their true burden.

In this review, we summarize the taxonomy, ecology, geographic range, clinical features, and epidemiology of *Coccidioides* species, *Histoplasma capsulatum*, *Blastomyces* species, *Paracoccidioides* species, and *Sporothrix* species in the Americas.

## 2. *Coccidioides* Species

Coccidioidomycosis, also known as valley fever, is a potentially serious fungal infection caused by two closely related fungi, *Coccidioides immitis* and *Coccidioides posadasii*. Infection is generally acquired by inhaling airborne asexual spores, arthroconidia, present in dust and soil [2]. Approximately 15,000 cases of coccidioidomycosis are reported to the Centers for Disease Control and Prevention (CDC) each year, but the estimated number of infections could be 6 to 14 times greater than the number of reported cases [3]. Approximately 60% of infected persons remain asymptomatic or develop mild symptoms, and the remaining 40% develop serious infection that often requires hospitalization [4]. Most serious cases manifest as severe pneumonia, and the infection can also disseminate to other organs. Mild cases are usually self-resolving and do not require treatment, whereas moderate and severe cases are treated with antifungals. The most dangerous manifestation of coccidioidomycosis is fungal meningitis, which is a potentially fatal disease that requires lifelong antifungal treatment [5].

*Coccidioides* species are found in regions with hot arid climates characterized by a prolonged dry season, with a short period of monsoon rains, and populated by the plant and animal communities belonging to the Lower Sonoran Life Zone [6]. For reasons not entirely understood, *Coccidioides* species are restricted to the American continents; most cases reported from other parts of the world are linked to travel to the Americas [7]. The ecological niche of these fungi is poorly understood. For a long time, *Coccidioides* was assumed to be a soil saprotroph [8]. However, recent genomic analysis identified amplification of gene families responsible for protein degradation [9], and environmental sampling identified a close association of this fungus with rodent habitats, which led to the hypothesis that *Coccidioides* sp. may depend on an animal host for propagation [10]. The role of desert animals and other ecological factors in the life cycle of *Coccidioides* is being investigated by several groups [11,12]. A better understanding of the ecological niche is important for understanding the current distribution and modeling the future expansion of this fungus.

*C. immitis* was divided into two closely related species, *C. immitis* and *C. posadasii,* in 2002 based on microsatellite marker analysis later confirmed by population genetics studies using whole-genome sequencing data [13,14,15]. In the United States, *Coccidioides* was historically believed to be restricted to the southwestern states. However, in the last two decades, *Coccidioides* has been found outside of these areas. For example, in 2001, an outbreak of coccidioidomycosis was reported among persons working at an archeological site in Dinosaur National Monument in northeastern Utah, and in 2014, DNA of both *C. posadasii* and *C. immitis* was detected in soils collected from this site [16]. In 2010, three cases of coccidioidomycosis were reported in southeastern Washington State in people without travel history in the known endemic areas [17], and in 2014, *C. immitis* was detected in soil at the potential exposure site by PCR and culture [18]. Whole-genome sequencing confirmed genetic identity between isolates from the patient and soil, confirming the presence of *C. immitis* in this area [18]. Since then, several locally acquired cases have been reported in Washington each year [19,20]. Notably, whole-genome sequencing of clinical and environmental isolates from Washington State detected an unusually close genomic relationship among isolates from different cases, which is indicative of the recent introduction of this fungus into this region [19]. Molecular evolutionary analysis estimates the introduction of *C. immitis* into Washington State in the last 100 years [13].

Aside from the newly identified endemic areas in Washington state, *C. immitis* primarily lives in California’s southern Central Valley. In contrast, *C. posadasii* lives in Arizona, Texas, and other southwestern states with a similar climate, as well as in Mexico and parts of Central and South America [4]. Over most of their ranges, the two species are genetically and geographically distinct, although both are found in Baja California, Mexico. Limited evidence of genetic introgression between the two species has been detected with genomic analysis [21]. While *C. immitis* and *C. posadasii* have distinctly different geographic ranges, no data exist to suggest that they cause disparate syndromes. Identification of *Coccidioides* to the species level is useful for epidemiological purposes, but it may not play a role in the course of treatment. Identification as *Coccidioides* species or *C. immitis/posadasii* is sufficient in the clinical microbiology laboratory (Table 1).

Habitat modeling is a useful tool for improving our understanding of the current and potential future distribution of coccidioidomycosis. Several predictive models show the likely expansion of suitable habitats for *Coccidioides* due to rising temperatures, which could lead to the spread of this fungus north and east of the current endemic areas [22,23,24,25,26]. The recent discoveries of *Coccidioides* sp. in Washington State and Utah support this prediction. Although the prevalence of coccidioidomycosis outside the United States is poorly understood, the same ecological and economic factors likely responsible for increasing incidence and geographic expansion of *Coccidioides* in the United States are likely affecting *C. posadasii* populations in Central and South America [27]. More research and better surveillance are needed to understand and deal with the emergence of this pathogen.

Maps that accurately depict the geographic distribution of *Coccidioides* sp. are essential for raising awareness among healthcare providers and the general public about the possibility of contracting coccidioidomycosis based on residence in or travel to certain geographic locations. Increased awareness among clinicians and the public can lead to faster diagnosis and treatment initiation [28,29]. The original and widely used maps describing the distribution of this fungus were drawn in the 1940s and were recently updated in 2020 [30].

In the United States, coccidioidomycosis is under public health surveillance in 26 states. In the last decade, the number of reported cases has increased [31,32]. The reasons for this recent increase are not well understood but could include increased awareness and improved diagnostics, rising temperatures, severe droughts, and increased urbanization [33,34]. Typically, Arizona reports the most coccidioidomycosis cases of any state. However, in 2018, California reported more cases for the first time since public health surveillance for coccidioidomycosis began [35].

## 3. *Histoplasma* Species

Histoplasmosis is caused by fungi in the genus *Histoplasma* [36]. *Histoplasma* was discovered and named in the Americas in 1906 by a clinician performing autopsies on patients presumed to have died of malaria in the Panama Canal Zone. It was incorrectly described as an encapsulated protozoon inside the cytoplasm of histiocytes, hence the name *Histoplasma capsulatum* [37]. Infection caused by *Histoplasma* begins in the lungs and can range from asymptomatic infection or localized nodules to mild respiratory symptoms to fulminant disseminated disease. Although *Histoplasma* is a primary pathogen and can cause infection in immunocompetent people, it is an AIDS-defining illness, especially in Latin America [38]. A common factor in *Histoplasma* outbreaks is aerosolization of spores after environmental disruption, often involving bird or bat guano [39]. Sporadic cases of histoplasmosis also occur and may also involve these types of exposures, though some cases are not clearly associated with a specific activity or source [40].

*Histoplasma* is endemic throughout the Americas, but the regions of endemicity are not clearly defined and may be larger than has been assumed in the past [30]. Within the United States, histoplasmosis is traditionally described as being endemic to the Ohio and Mississippi River valleys based on nationwide skin test surveys conducted during the late 1940s and early 1950s [41,42,43]. While histoplasmosis appears most commonly throughout the central and eastern United States, public health surveillance data and environmental modeling suggest an expanding focus of histoplasmosis to the northern parts of the United States [44,45]. Isolated case reports in humans and animals also suggest the potential for histoplasmosis to occur outside the traditionally described areas [46]. Based on these lines of evidence, CDC recently updated its map of the estimated range of *Histoplasma*, showing that *Histoplasma* is likely to live throughout most of the eastern half of the country and that it can potentially live throughout the western half of the country [30]. In Canada, *Histoplasma* is associated with the St. Lawrence River Valley in Ontario and Quebec, but it may be more widely distributed to the west [30,47,48]. *Histoplasma* is endemic throughout most of Latin America, the exceptions being the western half of Mexico and the western edge of South America, including coastal Peru and most of Chile [30]. *Histoplasma* lives throughout much of the Caribbean, and outbreaks have been reported with activities such as cave exploring on several islands [30,49,50] (Table 1).

A more comprehensive and detailed understanding of the geographic distribution of histoplasmosis could be made possible by additional environmental modeling studies and by improved public health surveillance. Currently, histoplasmosis is a reportable disease in only 12 U.S. states, which do not include certain states within the areas traditionally considered to be at the highest risk. In states where histoplasmosis is reportable, surveillance detects fewer than 1000 cases per year, whereas national hospital discharge data show that over 5000 histoplasmosis-related hospitalizations occur annually [44,51]. The difference between these two counts suggests that substantial under-reporting is occurring in the United States.

Recently, attempts have been made to define clades or cryptic species within *Histoplasma capsulatum*, aside from the Africa-associated *Histoplasma duboisii* and *Histoplasma farciminosum*, recognized as only causing disease in horses [52,53]. Kasuga and colleagues used multilocus sequence typing (MLST) to divide *Histoplasma* into eight clades, four of which were represented in the Americas [54]. Teixeira and colleagues expanded the MLST over a larger set of isolates and identified two clusters in the United States that likely represented unique species and four populations in Latin America that likely represented separate species [55]. In 2017, whole-genome sequencing was applied to multiple members of the genus *Histoplasma* for the first time, and four species were identified: *H. capsulatum sensu stricto* was restricted to isolates coming from Panama, the original site of discovery; *Histoplasma mississippiense* and *Histoplasma ohiense* represented the isolates from North America, but definitive geographic boundaries were not defined; and *Histoplasma suramericanum* represented the isolates from South America [56]. While the data supporting the division of *H. capsulatum* in the Americas into four distinct clades/species were compelling, the species designations did not strictly follow the rules of the International Code of Botanical Nomenclature and are currently considered to be invalid [57].

The division of *Histoplasma* in the Americas into separate clades or species is currently based on phylogenetic information only. No significant differences in the clinical course of infection have been described. As all species cause the same disease, differentiation of the species in the clinical laboratory may be useful for epidemiological purposes, but it does not contribute to the clinical course of treatment. Even if new names for the well-defined clades are established, a designation of *Histoplasma* species or *Histoplasma capsulatum* species complex will suffice for patient care.

Countries of the Americas have the goal of eliminating HIV as a public health problem by 2030 [58]. In Latin America, histoplasmosis is one of the most prevalent opportunistic infections affecting people living with HIV (PLHIV), with a high mortality rate of up to 30% [59,60]. A 2012 study estimated 6710 to 15,657 cases of histoplasmosis among PLHIV in Latin America each year [61]. In some countries, the prevalence and mortality associated with histoplasmosis are estimated to be equivalent to or higher than that of tuberculosis [62], indicating that histoplasmosis could be responsible for a significant proportion of the deaths each year in a few Latin American countries [62].

Some progress has been recently achieved, including the publication of World Health Organization (WHO)/Pan American Health Organization guidelines for diagnosis and management of disseminated histoplasmosis in PLHIV, the development, validation, and commercial availability of kits for rapid detection of *Histoplasma* antigen in human specimens, and the inclusion of commercially available diagnostic assays in the second WHO model list of essential in vitro diagnostics [63,64,65,66].

## 4. *Blastomyces* Species

Blastomycosis is a disease caused by fungi in the genus *Blastomyces* [67]. As with *Histoplasma* and *Coccidioides*, inhalation of conidia is the predominant mode of infection. More than 90% of the clinical cases are primary pulmonary infections, although many cases are asymptomatic [68,69]. Hematologic dissemination occurs in up to a quarter of symptomatic patients, and primary sites of dissemination include the skin, bone, genitourinary system, and central nervous system [70].

In a large study of blastomycosis patients in Wisconsin, hospitalization was two to three times more likely in Hispanic whites, American Indian/Alaska Natives (AI/AN), and Asians than for non-Hispanic whites, even though the former groups tended to be younger and healthier [71]. This is consistent with other reports showing unusually high case numbers among the Hmong ethnic group in Wisconsin and higher rates and mortality among blacks in Illinois and Missouri [72,73,74].

In the Americas, *Blastomyces* is endemic only to North America [30]. The known geographic range extends from the east coast of the United States [not including southern Florida] to the Mississippi River drainage in the west and from the gulf coast all the way into Ontario, Quebec, Manitoba, and Saskatchewan, Canada to the north [30]. Ecological niche modeling of *Blastomyces* around human and canine cases in Wisconsin identified proximity to waterways and moist soil were more predictive than soil characteristics, and one of the largest recorded outbreaks of blastomycosis occurred among boaters on the Little Wolf River [75,76]. Range expansion to the north has been spurred by case reports and case series in regions previously thought to be outside of the endemic zone [77,78,79] (Table 1).

Given the imprecise geographic range and the nonspecific signs and symptoms, misdiagnosis is a consistent problem with blastomycosis, which often leads to delays in appropriate treatment and increased mortality [69,78,79,80,81,82]. In a recent case series from New York, a newly defined endemic region, most cases were initially misdiagnosed as malignancy or a viral or bacterial infection [78].

Phylogenetic analysis of six genetically distinct loci has divided *Blastomyces* into two species, *Blastomyces dermatitidis* and *Blastomyces gilchristii* [83]. *B. gilchristii* appears to live primarily in the northern portion of the *Blastomyces* range around Wisconsin, Minnesota, and into Ontario, Canada. Interestingly, in a large case study of 477 patients in Wisconsin, 90% of the *B. dermatitidis* infections were among non-Hispanic whites, whereas the cases among Hispanic whites, Asians, and AI/AN were *B. gilchristii* [71]. In work that preceded the new nomenclature, *B. gilchristii* was more likely to cause pulmonary infection alone, whereas *B. dermatitidis* was more likely to cause disseminated infection [84]. Despite these differences, there are no recommendations for differential treatment of blastomycosis based on species, and the current Infectious Diseases Society of America treatment guidelines precede the discovery of the two distinct species. As for *Histoplasma* and *Coccidioides*, there are currently no clinical reasons for distinguishing between the two species of *Blastomyces*, but that may change as we learn more about the differences between them [85].

A new species, *Blastomyces helicus*, has been described from the Western United States and Canada [86]. This species was identified from sequence data of isolates identified as either *Blastomyces* or *Emmonsia* that came from the western United States and Canada, outside of the typical range of *Blastomyces*. Data are limited, but cases seem to occur mainly in immunocompromised persons and domestic animals (e.g., cats and dogs). The clinical course seems to be more severe than for *B. dermatitidis* or *B. gilchristii*. *B. helicus* can be distinguished from the other two species by histopathology, as the yeast cells tend to form chains and branches of elongate cells rather than round budding cells [86].

## 5. *Paracoccidioides* Species

Paracoccidioidomycosis (PCM) is caused by fungi in the genus *Paracoccidioides*, which are endemic in Latin America. The environmental reservoir of *Paracoccidioides* is unknown, but inhalation of contaminated soil or dust is believed to be the predominant mode of infection [87]. In adults, PCM begins as a pneumonitis but spreads through the lymphatic system to the lymph nodes, where it often forms granulomas and can become a continuous latent infection. A decrease in immunocompetency can lead to reactivation and hematogenous dissemination to multiple organs. Initial symptoms of reactivation are vague but consist of general malaise, anorexia, and weight loss. The chronic form is generally limited to lesions in the lung, oral mucosa, and on the skin around the mouth and nose. In children and young adults, a subacute infection can occur with symptoms of lymphadenomegaly, hepatosplenomegaly, and lesions of the skin and oral mucosa.

*Paracoccidioides* ranges from southern Mexico to northern Argentina. Although the northeastern limits of the range are Colombia and Venezuela, most cases (up to 80%) are recorded from south and southeast Brazil and northward [88]. The prevalence of PCM is low, with a range from 0.3–3 cases per 100,000 population in countries where the disease is endemic [87,89]. PCM is relatively uncommon in children, and the majority (~85%) of PCM cases occur in adults [87,89]. Active disease is more frequently observed in post-pubescent males than females (ratio of 13:1); estrogens, 17 beta-estradiol, confer disease protection in sexually mature women [90,91]. However, paracoccidioidin intradermal reactivity shows similar results between males and females [92]. Because it occurs most often in resource-limited settings and mimics tuberculosis in symptoms and course of infection, PCM is likely under-recorded [93] (Table 1).

The incubation period is highly variable, ranging from 1 month to decades. PCM cases diagnosed outside the endemic regions have been linked with prior visits to endemic regions [94,95]. Specific environmental characteristics associated with PCM cases include altitude from 1000 to 1499 m above sea level, annual rainfall from 2000 to 2999 mm, and the presence of humid forests and coffee and tobacco crops [96,97]. PCM is considered an occupational disease, as most cases occur among farmers and other persons regularly exposed to soil [98]. PCM outbreaks are rare but have been linked with occupational activities, for example, the construction of a highway in Rio de Janeiro, Brazil. Clusters have also been associated with the El Niño–Southern Oscillation climatic anomaly [99,100,101].

In 2014, Teixeira and colleagues used phylogenetic and morphologic analysis to separate isolates of *Paracoccidioides* from central and northern Brazil into the new species *Paracoccidioides lutzii* [102]. Although data suggest that the two species exhibit some differences in clinical course, there are no guidelines to suggest differential treatment. Identification of species is ideal for epidemiologic purposes, but for the clinical microbiology laboratory, a designation of *Paracoccidioides* species is sufficient for the initiation of optimal therapy.

In 2017, Turissini and coworkers used phylogenic data derived from multiple gene sequences as well as microsatellites to divide *P. brasiliensis* into four separate species; *Paracoccidioides americana*, *Paracoccidioides restrepiensis*, *Paracoccidioides venezuelensis*, and *Paracoccidioides brasiliensis sensu stricto* [103]. Although the phylogenetic data strongly suggest that the complex contains multiple species, the names are currently considered invalid due to not following the conventions of the International Code of Botanical Nomenclature [104].

## 6. *Sporothrix* Species

Sporotrichosis is a disease caused by fungi in the *Sporothrix schenkii* species complex, the most common species being *S. schenkii sensu stricto* [105]. *Sporothrix* as a genus is cosmopolitan and not truly endemic, but it shares the common trait of thermal dimorphism with the endemic fungi. The most common mode of infection is traumatic inoculation and is associated with contaminated plant debris, especially sphagnum moss [105]. Infection is generally a benign subcutaneous infection with lymphatic spread. In the United States, sporotrichosis appears to be most common in the south and south-central states but is a rare disease overall, with a rate of 2 cases per million population in a large nationwide sample of persons with commercial insurance [106]. As most patients with sporotrichosis presumably do not require hospitalization, sporotrichosis-associated hospitalizations are also uncommon, with an average annual rate of 0.35 hospitalizations per million persons during 2000–2013 and no apparent temporal trend during this time frame [107].

Comprehensive epidemiologic data about sporotrichosis in the United States are incomplete, and most information comes from outbreak investigations. These outbreaks have been primarily associated with sphagnum moss used in topiaries or as packing material for tree seedlings and with hay bales [108,109,110,111,112]. For unclear reasons, no published literature has described a U.S. sporotrichosis outbreak investigation since the late 1990s [108]. A true absence of sporotrichosis outbreaks in the last 30 years might reflect industry changes in harvesting or processing of plant materials most often associated with these outbreaks. Another explanation is that sporotrichosis is likely widely under-recognized since no routine public health surveillance exists for it. Therefore, our understanding of the true burden of sporotrichosis in the United States remains limited.

*Sporothrix brasiliensis* is both endemic and emerging. *S. brasiliensis* differs from other species within the *S. schenkii* species complex in that it is not acquired from the environment but rather almost exclusively through interactions with infected cats. *S. brasiliensis* is currently endemic primarily in Brazil, but there are some data indicating that *S. brasiliensis* cases are emerging in surrounding countries [113]. In humans, the disease is similar to that caused by *S. schenkii sensu stricto,* but is more likely to present with a papule or ulcer at the site of infection. Symptoms are similar in cats with ulcerated lesions, enlarged lymph nodes, and sometimes respiratory symptoms. The first outbreak of cat-transmitted *S. brasiliensis* disease was detected in 1998 in Rio de Janeiro, Brazil [113]. By 2020, cases of cat-transmitted sporotrichosis caused by *S. brasiliensis* have been reported in most coastal Brazilian states [113]. In Brazil, 4188 human cases were reported from 1997 to 2011, and 4703 cat cases were diagnosed through 2015, with a few additional cases in dogs [114,115,116]. As many infected cats are never captured and diagnosed, and symptoms in humans are not severe, the burden is likely much higher than reported. Confirmed cases of feline sporotrichosis caused by *S. brasiliensis* have been reported from Argentina, with outbreaks among humans in the Buenos Aires Province, and the El Calafate City, Santa Cruz Province, Southern Patagonia, and Paraguay, where cases were linked with sick cats introduced from Brazil [117,118,119].

*S. brasiliensis* has produced a significant increase in the incidence of zoonotic infection in humans and domestic animals [e.g., cats and dogs] in the last 20 years, alarming public health authorities across the Americas. In August 2019, the Pan American Health Organization released a regional alert on *S. brasiliensis* [120].

## 7. Conclusions

Endemic and other dimorphic fungi are present throughout much of the Americas. These fungi are a serious public health concern because they can be difficult to prevent, often present with symptoms that mimic those of other infections, and can be challenging to diagnose. In addition, endemic fungal diseases can cause severe infections in both previously healthy hosts and in persons with weakened immune systems, particularly due to advanced HIV disease. Avoiding endemic fungi in the natural environment is challenging. Therefore, prevention efforts are best directed at increasing awareness among healthcare providers and the public, encouraging patients to seek medical care, and helping clinicians order appropriate testing to enable faster diagnosis and treatment.

## Figures and Tables

**Table 1:** Summary of taxonomy, ecology, geographic range, clinical features, and epidemiology of coccidioidomycosis, histoplasmosis, blastomycosis, paracoccidioidomycosis, and sporotrichosis.

	Coccidioidomycosis	Histoplasmosis	Blastomycosis	Paracoccidioidomycosis	Sporotrichosis
Taxonomy	*C. immitis* and *C. posadasii*	*H. capsulatum* New species proposed, but classification currently not validated	In North America: *B. dermatitidis, B. gilchristii* and *B. helicus*	*P. brasiliensis* and *P. lutzii* New species proposed, but classification currently not validated	*Sporothrix species complex (S. schenckii, S. brasiliensis, S. globosa, S. mexicana, S. luriei, and* *S. pallida)*
Ecology	Hot arid regions, with prolonged dry season and short monsoon rainy season	Soil contaminated with Histoplasma, particularly soil contained bird or bat guano	Moist soil and decomposing organic matter such as wood and leaves	Unknown. Environmental characteristics include altitude from 1000 to 1499 m ASL, high annual rainfall, and presence of humid forests	Saprophytic of soil and plants. Found on a variety of mammals (especially cats)
Known geographic range	Western United States, northern regions of Mexico, Guatemala, Honduras, Venezuela, Brazil, Paraguay, and Argentina	Most countries in the Americas and the major islands in the Caribbean, except the western half of Mexico, the western coast of Peru, and most of Chile	In the Americas, primarily in North America (United States and Canada), especially areas surrounding the Ohio and Mississippi River valleys and the Great Lakes region	Restricted to Latin American countries, from Mexico to Argentina. Most cases from Brazil. Extremely rare in the Caribbean	Worldwide distribution, especially in tropical and subtropical regions. *S. brasiliensis* has been reported mostly in eastern Brazil, Argentina, and Paraguay
Clinical features	Pulmonary disease: acute respiratory infection, pulmonary nodules and cavities, and chronic fibrocavitary pneumonia Extrapulmonary disease: bones, joints, central nervous system	Acute and subacute pulmonary disease Disseminated disease (including acute, subacute, and chronic stages) Chronic pulmonary disease Disease sequelae *	Pulmonary disease: acute and chronic infection, and acute respiratory distress syndrome Extrapulmonary disease: skin, genitourinary, osseous, and central nervous system	Subclinical infection Residual form Acute/subacute disease Chronic progressive disease >90% of cases occur in males	Lymphocutaneous disease Multifocal extracutaneous disease Pulmonary disease (rare)
Epidemiology	Approximately 15,000 cases reported per year in the United States, mainly from Arizona and California	Approximately 1000 cases reported from 12 U.S. states annually, but the true burden is unknown. In PLHIV from LATAM was estimated 6710–15,657 cases (2020)	In U.S. states where disease is reportable, incidence rates 1 to 2 cases per 100,000 population. Hyperendemic regions rates ranging from 10 to 40 cases per 100,000 persons	Most cases reported in Brazil (~80%). In endemic countries, estimated prevalence ranges from 0.3–3 cases per 100,000 population	Estimated 2 cases per million population in the United States

(PLHIV) people living with HIV, (CNS) central nervous system, (Ag) antigen, (CDC) Centers for Disease Control and Prevention, (LATAM) Latin America countries, (ASL) above sea level, (*) fibrosis and antigen hypersensitivity.
